# Effects of Letrozole Treatment and Vitamin C Supplementation on Morphology, Endoplasmic Reticulum Stress, Programmed Cell Death, and Oxidative Stress in the Small Intestine of Adult Male Rats

**DOI:** 10.3390/cimb46030127

**Published:** 2024-03-01

**Authors:** Anna Pilutin, Sylwia Rzeszotek, Aleksandra Wilk, Klaudia Klimaszewska, Julia Łukasiewicz, Rufaro Lynnette Mafuta, Thanushan Nagendran, Rupia Ndambara, Barbara Wiszniewska

**Affiliations:** Department of Histology and Embryology, Faculty of Medicine and Dentistry, Pomeranian Medical University, Powstańców Wlkp. 72 Str., 70-111 Szczecin, Poland; sylwia.rzeszotek@pum.edu.pl (S.R.); klaudia.klimaszewska@op.pl (K.K.);

**Keywords:** estrogens, male, small intestine, aromatase, letrozole, morphology, endoplasmic reticulum stress, oxidative stress

## Abstract

Estrogens are hormones that play an important role in the digestive tract, including in men. Letrozole is an inhibitor of cytochrome P450 aromatase, an enzyme converting androgens to estrogens. The use of letrozole may cause oxidative stress and endoplasmic reticulum stress in the cells. Factors modulating cellular stress may include vitamin C. The purpose of this study was to examine whether letrozole and/or vitamin C supplementation can affect the morphology of the small intestine, the parameters of endoplasmic reticulum stress, programmed cell death markers, and oxidative damage. Three-month-old male rats were divided into four groups and treated with the following: (I) CTRL—water; (II) CTRL+C—L-ascorbic acid; (III) LET—letrozole; and (IV) LET+C—letrozole + L-ascorbic acid. The morphometrical measurements included epithelial thickness, crypt and lumen area, crypt perimeter, nuclei number in the crypt, and the cell size of crypts. The expression levels of PERK, caspase-3, and catalase were determined. Significant differences in the morphometrical measurements and immunoexpression were observed. This may indicate that chronic treatment with letrozole can affect morphology and induce ER stress, oxidative stress, and programmed cell death in the epithelial cells of the small intestine of adult male rats. Vitamin C supplementation exerts an effect on some parameters of the molecular processes.

## 1. Introduction

In recent years, it has been shown that estrogens (ESTs) and their receptors (ERs) play an important role in the proper functioning of the gastrointestinal tract in both males and females. EST imbalance contributes to the development of a number of pathological conditions, including gastroesophageal reflux; cancers of the esophagus, stomach, and colon; gastric ulcers; inflammatory bowel disease; or irritable bowel syndrome [1]. The most potent estrogens are synthesized by the aromatization of androstenedione and testosterone. 

Letrozole (LET) is a nonsteroidal inhibitor of cytochrome P450 aromatase, an enzyme converting androgens to estrogens [2]. Earlier studies conducted by our team showed that the long-term administration of LET to male rats led to morphological changes similar to the aging characteristics of the gonads [3,4], epididymis [5], and bone tissue [6], as well as changes in the number of apoptotic cells [5]. Additionally, a review of the scientific literature suggests that estrogen deficiency may cause oxidative stress (OS) and endoplasmic reticulum stress (ERET Stress) in cells [7,8,9,10]. OS and ERET Stress are commonly observed in the tissue cells of the jejunum and may contribute to the development of pathological conditions. Moreover, in gastrointestinal tissues, EST can modulate the stress response of the endoplasmic reticulum [11].

Oxidative stress is defined as a disturbance in the balance between the production of reactive oxygen species (free radicals) and antioxidant defenses. The body has evolved major antioxidant defense mechanisms to protect it from free radical attack. These defenses can be conveniently considered as cellular, membrane, and extracellular mechanisms. Cellular antioxidant defenses include the dismutase, peroxidase, and catalase enzymes [12]. A catalase is one of the crucial antioxidant enzymes that mitigates oxidative stress to a considerable extent by destroying cellular hydrogen peroxide. The deficiency or malfunction of catalase is postulated to be related to the pathogenesis of many age-associated degenerative diseases like diabetes mellitus, hypertension, anemia, vitiligo, Alzheimer’s disease, Parkinson’s disease, bipolar disorder, cancer, and schizophrenia [13].

The endoplasmic reticulum (ERET) is a membranous intracellular organelle and the first compartment of the secretory pathway. It contributes to the production and folding of approximately one third of cellular proteins and is thus inextricably linked to the maintenance of cellular homeostasis and the fine balance between health and disease [14]. ERET is sensitive to many variables, such as changes in Ca2+ level, ERET stressor molecules, temperature, reactive oxygen species (ROS), local and temporary decreases in energy (glucose) or oxygen levels, or the accumulation of misfolded proteins. In response to stressors, ERET activates the mechanism that adapts/adjusts the organelles and cells to a stressful situation. The three main ERET Stress signaling pathways, IRE-1, PERK, and ATF6, are kept inactive by a heat shock protein chaperone, also called GRP78, in the ERET membrane. Triggering the signaling cascade under stress starts a powerful arsenal of signaling molecules and is called the unfolded protein response (UPR) [14,15].

One factor that reduces the effects of OS and modulates ERET Stress in cells and tissues may be supplementation with the antioxidant factor—vitamin C [16,17]. Therefore, we hypothesized that vitamin C may have a protective effect against letrozole treatment. The aim of our study was to examine whether LET and LET administered in combination with vitamin C can affect the morphology of the jejunum and to determine whether LET and LET administered in combination with vitamin C can change certain parameters of ERET Stress, programmed cell death markers, and oxidative damage.

## 2. Materials and Methods

The experiment was conducted in full accordance with Polish Law and with the approval of the ethics committee of the Pomeranian Medical University in Szczecin (approval no. 23/2017). Sexually mature 3-month-old male Wistar rats were randomly divided into four groups, as shown in Table 1: Control (CTRL), Control supplemented with vitamin C (CTRL+C), Letrozole (LET), and Letrozole supplemented with vitamin C (LET+C). Each group consisted of 6 rats. 

Rats in the LET group received Letrozole, a non-steroidal inhibitor of cytochrome P450 aromatase, orally at a dose of 1 mg/kg b.w./day for 6 months (Femara, Novartis, Basel, Switzerland). LET was given to each experimental rat once per day in the morning in the form of a small pellet made from LET powder and pressed into a piece of bread. The animals willingly ate the pellets from the hand of the person conducting the experiment. 

The rats in the CTRL group received a pellet without LET. The rats in the CTRL+C group received water with vitamin C (Ascorgem, Karczew, Poland) at a dose of 500 mg/L [18]. The rats in the LET+C group received a pellet with LET and water supplemented with vitamin C (Table 1).

At the end of the experimental treatment, the animals were sacrificed under thiopental anesthesia following the approved protocol. The jejunums were collected, fixed in formalin, and embedded in paraffin. A series of sections (3–5 μm) were prepared from the paraffin-embedded tissues.

For the morphological and morphometrical analyses, dewaxed and hydrated sections were stained using the hematoxylin and eosin methods. Histological measurements were made using the LAS imaging analysis software under a light Leica microscope. The epithelial thickness was measured from the basement membrane to the apical surface of the epithelium. For each animal, 50 locations were measured in a cross-section of the jejunum. A lens magnification of 40x was used.

In addition, 50 crypts were measured for each animal in a cross-section of the jejunum. Measurements were made on cross-sections of crypts most similar to a circle. The following measurements were taken: crypt area, crypt perimeter, crypt lumen area, and nuclei number. The average cell size was determined. This was carried out by subtracting the crypt lumen area from the total crypt area and then dividing the resulting value by the nuclei number. The nuclei number represents the number of mucosal cells present on each transverse image of the crypt. All crypt measurements followed the published protocol [19]. The measurements taken were as follows: crypt area (CA, in µm2), crypt perimeter (CP, in µm), crypt lumen area (LA, in µm2), and nuclei number (NN). The average cell size (CS, in µm^2^) was calculated as CS=(CA-LA)/NN.

To identify the parameters of ERET Stress, programmed cell death markers, and oxidative damage, specific antibodies and immunohistochemistry reactions were employed using the EnVision system (Agilent, Santa Clara, CA, USA). The slides were incubated with primary antibodies at room temperature (RT) for 1 h. 

The antibody used to identify ERET Stress was mouse monoclonal anti-PERK antibody, Santa Cruz Biotechnology (Dallas, TX, USA), cat no:sc-377400; dilution 1:200. 

The antibody used to identify oxidative damage was mouse monoclonal anti-catalase antibody, Santa Cruz Biotechnology (Dallas, TX, USA), cat no:sc-365738; dilution 1:200. 

The antibody used to identify apoptotic cells was mouse monoclonal anti-caspase-3 antibody, Santa Cruz Biotechnology (Dallas, TX, USA), cat no:sc-56053; dilution 1:200.

The antibodies were diluted in Antibody Diluent (cat# ab64211, abcam, Cambridge, UK). After washing, the slides were covered with ready-to-use EnVision FLEX LINKER, for 30 min at RT. To visualize the antigen–antibody complex, a reaction of avidin–biotin–horseradish peroxidase with DAB as a chromogen was performed, following the included staining procedure instructions. The sections were washed in distilled H_2_O and counterstained with hematoxylin. Negative controls were processed without the primary antibody. Positive and negative stainings were determined by visual identification of brown pigmentation using a microscope (Leica DM5000B, Wetzlar, Germany).

To facilitate digital image analysis, stained slides were scanned using a 3DHISTECH Pannoramic MIDI II scanner (Sysmex Polska Sp. z o.o. Warsaw, Poland), with a lens magnification of 20x, resulting in images with a resolution of 0.17 µm/pixel. The Pattern Quant software from 3DHISTECH (3DHISTECH Kft. Budapest, Hungary) was utilized for digital image analysis. This module enabled the initial segmentation of tissues and the identification of various tissue structures, based on characteristics such as structure, size, color, and color intensity.

**Figure 1:**
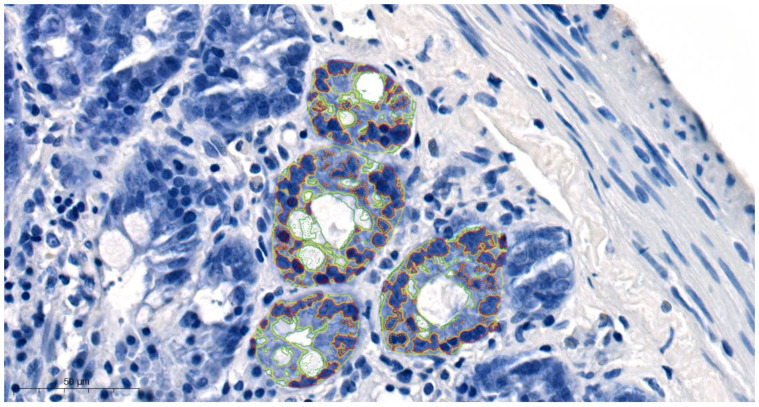
Quantitative digital image analysis created by Pattern Quant. Red circles—cell nuclei; green circles—goblet cells; yellow circles—cytoplasm.

Quantitative digital image analysis was employed to assess small intestinal crypt cells. Parameters such as area, perimeter occupied by the assessed structure, and the intensity of staining were determined. Due to the fact that Pattern Quant is a software that visualizes different structures that are characterized by both (i) pattern and (ii) color, we were able to examine cell nuclei, goblet cells, and cytoplasm. We trained the program to recognize the tested structures. We divided separated structures into the following clusters: red circles—cell nuclei; green circles—goblet cells; yellow circles—cytoplasm (Figure 1). The results obtained by the Pattern Quant were expressed as values (counts). Additionally, the obtained results were calculated as the percentage of cell nuclei, goblet cells, and cytoplasm and presented as bar graphs. All calculations were analyzed by two independent experts. The obtained results underwent statistical analysis.

The results were analyzed statistically using Statistica 6.1 software (StatSoft, Kraków, Poland). Statistical significance was determined using the analysis of variance (ANOVA) test, and a multiple comparison post test was employed to evaluate the differences between the studied groups. Medians, arithmetical means, and standard deviations (±SD) were determined for each parameter.

## 3. Results

### 3.1. Morphology

A light microscopy examination of all groups revealed the four-layer structure of the intestinal wall, including the tunica mucosa, tunica submucosa, tunica muscularis, and tunica serosa. The tunica mucosa was entirely covered with a simple columnar epithelium, and scattered goblet cells were observed between the epithelial cells. Villi, finger-like projections arose from the mucosa, and simple tubular Lieberkühn glands (crypts) were observed at the base of the villi in the lamina propria. The submucosa consisted of connective tissue, while the tunica muscularis comprised inner circular and outer longitudinal layers of smooth muscle cells. The tunica serosa was formed by connective tissue.

### 3.2. Morphometry

**Figure 2:**
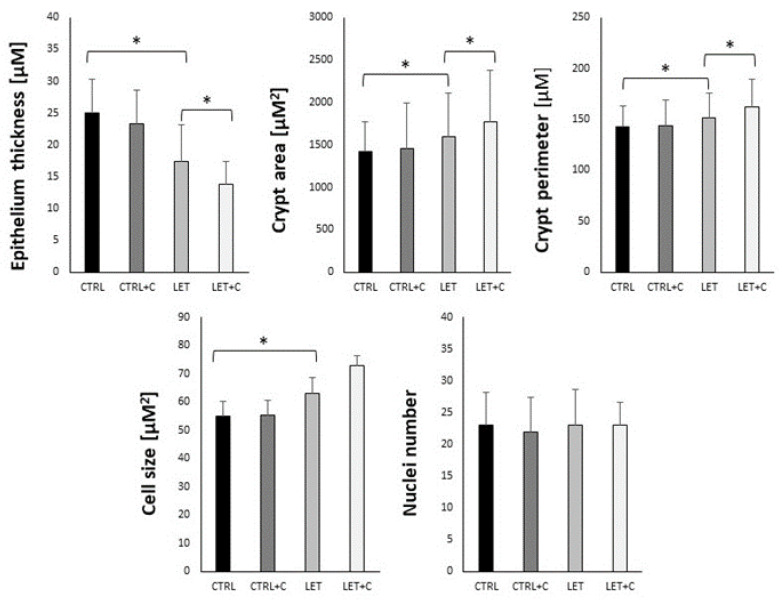
Histograms representing the morphometric measurements comparing data from all experimental groups. CTRL—control, CTRL+C—control group supplemented with vitamin C, LET—letrozole-treated group, LET+C—letrozole-treated and vitamin C-supplemented group. The data are presented as medians (Me). An asterisk (*) indicates statistically significant differences between groups (*p* < 0.05).

The intestinal epithelium thickness tended to be smaller in the LET and LET+C groups compared to the CTRL group. These differences were statistically significant (Table 2). The total area and perimeter of the crypt, as well as cell size, were larger in the LET and LET+C groups than in the CTRL and CTRL+C groups. These differences were statistically significant. However, the nuclei number did not differ significantly among the groups (Figure 2, Table 2).

### 3.3. IHC Staining

**Figure 3:**
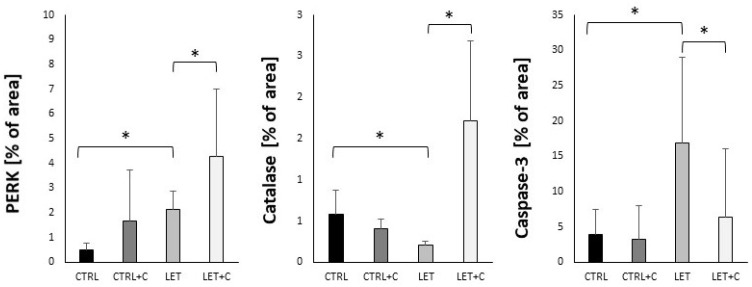
Histograms representing the immunohistochemical analysis comparing data from all experimental groups. CTRL—control, CTRL+C—control group supplemented with vitamin C, LET—letrozole-treated group, LET+C—letrozole-treated and vitamin C-supplemented group. The data are presented as medians (Me). An asterisk (*) indicates statistically significant differences between groups (*p* < 0.05).

**Figure 4:**
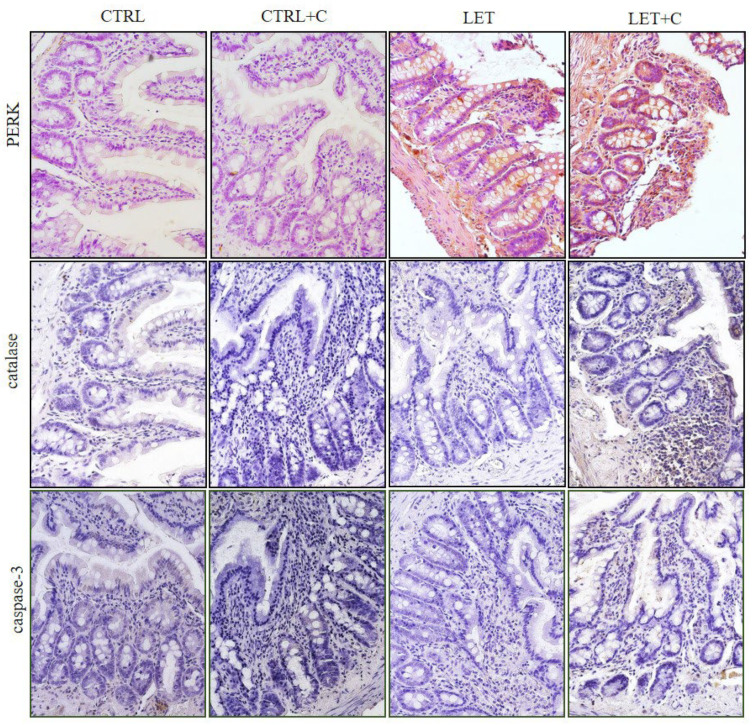
The effects of immunohistochemical staining with antibodies against PERK, catalase, and caspase-3 in all experimental groups. CTRL—control, CTRL+C—control group supplemented with vitamin C, LET—letrozole-treated group, LET+C—letrozole-treated and vitamin C-supplemented group.

Statistically significant differences were observed in the immunoexpression of PERK, catalase, and caspase-3 between the CTRL and LET groups, as well as between the LET and LET+C groups (Figure 3 and Figure 4; Table 2).

### 3.4. Digital Analysis

There were statistically significant differences in the total area of nuclei in the crypt in the LET and CTRL+C groups compared to the CTRL group. There were no statistically significant differences in the total area of nuclei between the LET+C and LET groups. 

There were statistically significant differences in the total area of cytoplasm in the crypt in the LET and CTRL+C groups compared to the CTRL group, as well as in the LET+C group compared to the LET group. 

**Figure 5:**
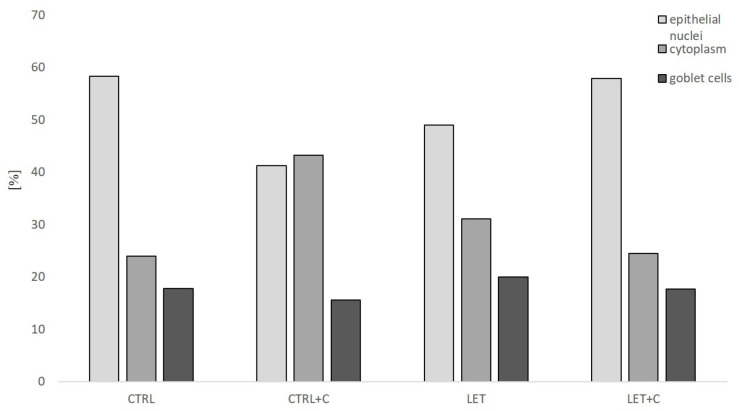
Percentage differences in epithelial nuclei, cytoplasm, and goblet cell area in the experimental groups evaluated through digital analysis by Pattern Quant.

There were statistically significant differences in the total area of goblet cells cytoplasm in the crypt in the LET and CTRL+C groups compared to the CTRL group. There were no statistically significant differences in the total area of nuclei between the LET+C and LET groups (Figure 5).

## 4. Discussion

In this study, we investigated the effects of letrozole (LET) treatment, with or without vitamin C, on morphological and morphometrical changes, as well as selected markers of ERET Stress and apoptosis in the jejunum of rats. Our previous studies demonstrated that the long-term administration of LET to male rats resulted in morphological changes similar to those observed in aging reproductive tissue [3,4,5] and aging bone tissue [6]. Based on these findings, we hypothesized that LET administration may induce OS and ERET Stress. To explore the potential protective effects, we selected vitamin C as an antioxidant since it is involved in various biological processes and acts as a cofactor for different metabolic enzymes [20]. 

In recent years, there has been an increasing focus on the influence of EST and ER on digestive system diseases. For example, a reduced level of EST has been associated with an increased risk of gastroesophageal reflux disease (GERD) [1], and gender differences have been observed in esophageal cancer [11]. This is particularly important as EST levels in men largely depend on local synthesis. Notably, studies conducted in vitro and in vivo by Wang et al. [11] have shown that estradiol treatment inhibits the viability and migration of esophageal cancer cells through the activation of ERET Stress [11]. It is important to mention that the observed effects of EST depend on the intensity and duration of stress conditions, leading to either the promotion of repair mechanisms or, in cases of prolonged or intense cellular stress, the activation of apoptosis pathways.

The compact and functional epithelial layer, with the proper expression of surface molecules, serves as a crucial protective and nourishing component of the body. Although epithelial cells adhere to one another and express cell adhesion molecules that are connected to the cytoskeleton, they are highly dynamic and undergo changes such as the invagination, elongation, and separation of epithelial cell sheets. Recent studies have highlighted the role of changes in the cell height associated with mitosis and apoptosis in contributing to epithelial invagination [21]. 

Vitamin C, also known as ascorbic acid, is a water-soluble antioxidant and enzyme cofactor present in plants and some animals. Most mammals and humans do not have the ability to synthesize this nutrient endogenously and, therefore, obtain it through diet. Vitamin C is the most commonly used vitamin and it takes part in many biochemical processes in organisms. It has different properties including anticancer, antioxidant, anti-aging, and immunity-enhancing properties [20]. Vitamin C has been described as a stimulator of stem cell and T lymphocyte proliferation, as well as an inhibitor of the migration and proliferation of human lens epithelial cells, thereby reducing the risk of cataracts [22]. However, limited information is available regarding the impact of estrogen and vitamin C imbalances on the epithelium of the jejunum. Since the number of cell nuclei within the intestinal crypts did not differ significantly, it can be concluded that disturbances in estrogen metabolism do not have a significant effect on the proliferative potential of intestinal epithelial cells. However, there was an increase in crypt area and perimeter, which was observed in the LET group and further enhanced when LET was combined with vitamin C. Interestingly, we also observed a concurrent reduction in the epithelium height, along with an increase in the area occupied by cytoplasm and goblet cells in the LET group compared to the CTRL group. These observations are consistent with the work of Kondo and Hayashi [21], which suggests that basal expansion accompanying cell shortening leads to larger tissue curvature. Kondo and Hayashi [21] proposed the involvement of microtubules in the changes in cell height and width of the apical and basal parts of cells, which, in cylindrical cells, are arranged parallel to the long axis of the cell, although the precise mechanisms regulating their functions remain unknown. In our work, the causes of changes in the height of the epithelium, crypt area, and perimeter could perhaps also be seen in changes in the proper functioning of cytoskeletal elements. As early as 1995, it was noted that certain compounds with estrogenic activity, such as 170-estradiol (E2), could cause damage to the kinetochore, the site where microtubules (MTs) attach [23]. This damage can lead to an increased risk of impaired cell division and changes in the karyotype. It has been known for a long time that the effects of estrogens on microtubule function can vary depending on the dose [24]. For instance, 17α-estradiol, which is less hormonally active and non-carcinogenic, can have an impact on MTs comparable to diethylstilbestrol (DES) or E2 [21,25]. Another explanation of the changes in the height of the epithelium, crypt area, and perimeter, closely related to cytoskeletal elements, can be adhesion molecules. Compelling evidence for this hypothesis is provided by Wada-Hiraike et al. [26]. According to them, the destabilization of cellular integrity and cytoarchitecture is observed as a consequence of α-catenin and plectin decrease and malpositioning, especially of the epithelium of the colonic tissue. Therefore, our results can be considered as preliminary and encourage further studies to support observed associations. 

It is noteworthy that the area occupied by cell nuclei, cytoplasm, and goblet cells in the crypts of the LET+C group was similar to that of the CTRL group. This suggests that vitamin C played a protective role in relation to the administration of an aromatase inhibitor. However, it is intriguing that significant changes in these parameters were caused by vitamin C supplementation alone. Similar results were reported by Amer et al. [27], who found a significant decrease in the goblet cell count in the duodenum and jejunum of broiler chickens following dietary vitamin C supplementation. In both cases, the role of vitamin C can be emphasized due to its involvement in blocking oxidative damage in cells.

On the other hand, high concentrations of vitamin C can act as a pro-oxidant inducing OS, either by generating reactive oxygen species or by inhibiting the antioxidant systems in the presence of iron, which, in turn, induces lipid peroxidation [28]. Increased OS can also lead to autophagy formation in cancer cell death. Therefore, the induction of autophagy may be mediated by vitamin C [29]. 

Our findings regarding the level of the PERK protein suggest that both estrogen metabolism disorders and the mechanism of action of vitamin C can initiate a cellular stress response. The elevated expression of the PERK protein in the ERET indicates an attempt to activate mechanisms that adapt the organelles and cells to stressful conditions. The research of Chimento et al. [30] suggested that breast cancer cells, following a prolonged period of estrogen deprivation, can adapt to low estrogen levels by enhancing their sensitivity to E2 [30]. It is possible that the conditions created in our experiment resulted in a cellular response manifested primarily by changes in the expression of the PERK protein. It is important to note that the unfolded protein response (UPR) represents a collection of adaptive signaling pathways that have evolved to address protein misfolding and restore an efficient protein-folding environment [9].

It is well known that steroid hormones, including estrogens, have a protective effect against apoptosis [31]. Estrogen deprivation in neuronal, endothelial, and testicular cells has been shown to induce apoptosis [32,33,34]. In our study, we observed a significant increase in the intensity of caspase-3 expression in the epithelial cells of rats in the LET group. The morphometric and morphological changes we observed may be related to this observation, as the apoptotic pathway activates the executioner caspase (e.g., 3 or 7), resulting in nuclear and cytosolic morphological changes [30,35]. However, when vitamin C supplementation was combined with letrozole treatment, the expression of caspase-3 decreased compared to the LET group. Apoptosis can be induced in cells under various conditions [36], and oxidative stress is known to be one of its triggers [37]. We hypothesize that the reduction in estrogen levels may contribute to the occurrence of oxidative stress in our experimental model. Our results suggest that this effect can be partially mitigated by supplementation with antioxidants such as vitamin C.

The gradual loss of estrogen, e.g., during menopause, is associated with a decrease in the level of antioxidant enzymes [38]. In our model, we observed a statistically significant decrease in catalase expression in the letrozole-treated group compared to the control group. A reduction in catalase activity, as a result of letrozole administration to animals, was also observed by Sushma et al. [39] and Pandev et al. [40]. We observed a statistically significant increase in catalase expression in the LET+C group compared to the LET. The effects of vitamin C on the level of antioxidant enzyme activity are most likely closely dependent on estrogen levels; for example, reduced antioxidant activity was observed in adipose tissue stem cells obtained from perimenopausal women. Increasing catalase expression in postmenopausal cells restored estrogen receptor (ER) expression [38]. It is worth noting that the metabolism of vitamin C is very complex. There are numerous polymorphisms of genes responsible for sodium-dependent vitamin C transport, and the observed effects depend on the dose used. Vitamin C is classified as an antioxidant that scavenges free radicals;, it also has a "second face" as a pro-oxidant factor, but this does not necessarily mean harmful effects at the cellular level [41]. Vitamin C usually acts as a catalase inhibitor, but in the event of a disturbed estrogen balance, its effect may be changed. There are known observations of the relationship between the protective effect of catalase dependent on estrogens, e.g., in the case of coronary diseases [42]. Studies conducted on a group of women showed a correlation between the level of catalase in red blood cells and the concentration of estrogens in the plasma [43]. Unfortunately, there is a lack of consistency in the results in this area.

## 5. Conclusions

Chronic treatment with letrozole can affect the morphology of the jejunum, including epithelial thickness and crypt structure, and induce endoplasmic reticulum stress and oxidative stress in adult male rats. Vitamin C supplementation, in conjunction with a non-steroidal aromatase inhibitor, can influence the thickness of the intestinal epithelium and crypt area and circumference, but it does not affect cell size. The protective effect of vitamin C supplementation against endoplasmic reticulum stress in the presence of non-steroidal aromatase inhibitors may vary, although our findings indicate that vitamin C supplementation reduced the number of apoptotic cells. Therefore, when considering vitamin C supplementation for patients undergoing letrozole treatment, the potential benefits and drawbacks should be carefully evaluated. These findings raise the question of whether ascorbic acid intake during letrozole treatment could be beneficial.

## Figures and Tables

**Table 1:** Experimental groups. The treatment lasted 6 months. N = 6.

No.	Group	Description	Treatment
I	CTRL	Control group	A bread pellet
II	CTRL+C	Control group supplemented with vitamin C	A bread pellet + water with vitamin C, at a dose of 500 mg/L of water
III	LET	Letrozole-treated group	A bread pellet with LET at a dose of 1 mg/kg b. w./day
IV	LET+C	Letrozole-treated and vitamin C-supplemented group	A bread pellet with LET at a dose of 1 mg/kg b. w./day + water with vitamin C at a dose of 500 mg/L

**Table 2:** A summary of the morphometric measurements and immunohistochemical analysis comparing data from all experimental groups.

	Groups			
	CTRL	CTRL+C	LET	LET+C
**Epithelium thickness [µm]** X ± SD Me	25.18 ± 5.25 25.01	22.95 ± 5.38 23.29	18.19 ± 5.72 17.42 *	14.59 ± 3.62 13.78 ^#^
**Crypt area [µm^2^]** X ± SD Me	1462.35 ± 347.68 1418.367	1523.86 ± 533.43 1459.98	1651.31 ± 519.98 1592.32 *	1867.65 ± 608.13 1771.39 ^#^
**Crypt perimeter [µm]** X ± SD Me	144.48 ± 20.63 142.82	145.22 ± 26.05 143.28	153.32 ± 25.14 151.06 *	162.97 ± 27.35 162.28 ^#^
**Cell size [µm^2^]** X ± SD Me	55.97 ± 17.4 54.84	62.46 ± 29.50 55.37	69.37 ± 23.91 62.90 *	76.74 ± 26.59 72.73
**Nuclei number** X ± SD Me	23.9 ± 4.02 23.00	23.16 ± 3.79 22.00	23.86 ± 4.11 23.00	23.90 ± 4.71 23.00
**PERK** **[% of area]** X ± SD Me	0.58 ± 0.24 0.51	2.34 ± 2.08 1.67	2.40 ± 0.72 2.15 *	4.77 ± 2.73 4.28 ^#^
**Catalase** **[% of area]** X ± SD Me	0.67 ± 0.29 0.58	0.41 ± 0.12 0.40	0.22 ± 0.05 0.21 *	1.79 ± 0.97 1.71 ^#^
**Caspase-3** **[% of area]** X ± SD Me	4.56 ± 3.56 3.91	4.48 ± 4.76 3.26	18.75 ± 12.13 16.89 *	9.19 ± 9.71 6.33 ^#^

The data are presented as mean (X), standard deviation (SD), and median (Me). An asterisk (*) indicates statistically significant differences compared to the control group (CTRL) (* *p* < 0.05 vs. CTRL), while a hashtag (#) indicates statistically significant differences compared to the letrozole-treated group (LET) (# *p* < 0.05 vs. L).

## Data Availability

The data presented in this study are available on request from the corresponding author.
